# Conserved Subgroups of the Plant-Specific RWP-RK Transcription Factor Family Are Present in Oomycete Pathogens

**DOI:** 10.3389/fmicb.2020.01724

**Published:** 2020-07-28

**Authors:** Maozhu Yin, Zhichao Zhang, Mingrun Xuan, Hui Feng, Wenwu Ye, Xiaobo Zheng, Yuanchao Wang

**Affiliations:** ^1^Department of Plant Pathology, Nanjing Agricultural University, Nanjing, China; ^2^Key Laboratory of Integrated Management of Crop Diseases and Pests, Ministry of Education, Nanjing, China; ^3^The Key Laboratory of Plant Immunity, Nanjing Agricultural University, Nanjing, China

**Keywords:** transcription factor, evolution, plant, oomycete, nitrate signaling

## Abstract

Nitrogen is a major constituent of proteins, chlorophyll, nucleotides, and hormones and has profound effects on plant growth and productivity. RWP-RK family transcription factors (TFs) are key regulators that bind to *cis*-acting elements in the promoter regions of nitrogen use efficiency-related genes and genes responsible for gametogenesis and embryogenesis. The proteins share a conserved RWPxRK motif; have been found in all vascular plants, green algae, and slime molds; and are considered to be a plant-specific TF family. In this study, we show that RWP-RK proteins are also widely present in the Stramenopila kingdom, particularly among the oomycetes, with 12–15 members per species. These proteins form three distinct phylogenetic subgroups, two of which are relatively closely related to the nodule inception (NIN)-like protein (NLP) or the RWP-RK domain protein (RKD) subfamilies of plant RWP-RK proteins. The donor for horizontal gene transfer of RWP-RK domains to slime molds is likely to have been among the Stramenopila, predating the divide between brown algae and oomycetes. The RWP-RK domain has secondary structures that are conserved across plants and oomycetes, but several amino acids that may affect DNA-binding affinity differ. The transcriptional activities of orthologous RWP-RK genes were found to be conserved in oomycetes. Our results demonstrate that RWP-RK family TF genes are present in the oomycetes and form specific subgroups with functions that are likely conserved. Our results provide new insights for further understanding the evolution and function of this TF family in specific eukaryotic organisms.

## Introduction

Nitrogen is a major constituent of proteins, chlorophyll, nucleotides, and hormones and has profound effects on plant growth and productivity (Crawford, 1995; Gojon, 2017). In the green alga *Chlamydomonas reinhardtii*, vegetative cells differentiate into gametes in response to nitrogen starvation, and the minus and plus programs of gametic differentiation are switched on and switched off, respectively, by the minus dominance (MID) protein, which is a transcription factor (TF). This protein contains an RWPYRK sequence that went unnoticed when the protein was initially identified, but it was the first identified member of what would be later described as RWP-RK TFs (Ferris and Goodenough, 1997). Subsequently, the first nodule inception (NIN) protein was identified in the legume plant *Lotus japonicus* as a crucial regulator, which controls nitrogen-mediated symbiotic root nodule formation. Sequence comparison between the NIN and MID proteins identified a conserved RWP-RK domain that is involved in DNA binding. Proteins containing this conserved domain encompassing the RWPXRK motif were then named RWP-RK proteins and defined as a new class of TFs (Schauser et al., 1999).

In recent years, genome-wide identification of RWP-RK proteins or NIN-like proteins (NLPs) has been conducted in many plant species (Schauser et al., 2005; Koi et al., 2016; Ge et al., 2018; Kumar et al., 2018; Liu et al., 2018; Wang Z. et al., 2018; Mu and Luo, 2019). Thus far, the RWP-RK protein family has been found in all vascular plants, green algae, and slime molds and is considered to be a plant-specific TF family (Mu and Luo, 2019). In addition, two sub-families have been classified; i.e., NLPs and RWP-RK domain proteins (RKDs). The proteins in both sub-families share the RWP-RK domain; however, NLPs carry an additional C-terminal region Phox and Bem1 (PB1) domain, an octicosapeptide that allows interaction with other proteins, and an additional N-terminal region nitrate responsive domain (NRD) that allows NLPs to receive nitrate signals (Chardin et al., 2014; Mu and Luo, 2019).

The RWP-RK proteins bind to *cis*-acting elements in the promoter regions of nitrogen use efficiency (NUE)-related genes (including nitrate reductase *NIA1* and nitrite reductase *NIR1*) and the genes responsible for gametogenesis and embryogenesis (Konishi and Yanagisawa, 2013a). In general, NLPs regulate tissue-specific expression of genes involved in NUE (Konishi and Yanagisawa, 2013b; Yu et al., 2016), while RKDs regulate expression of genes involved in gametogenesis or embryogenesis (Lin and Goodenough, 2007; Koi et al., 2016). In addition, the NRD domain in the N-terminal region of NLPs can respond to nitrate signals and bind specifically to nitrate responsive elements (NREs) found in the promoter regions of nitrate inducible genes (Castaings et al., 2009; Konishi and Yanagisawa, 2013a).

Oomycetes form a diverse group of eukaryotic microbes that outwardly resemble fungi in their growth habits and nutritional strategies, but are actually classified in the kingdom Stramenopila and are more closely related to golden-brown algae, diatoms, and brown algae (Sogin and Silberman, 1998). Many oomycetes are saprophytes or pathogens not only of plants but also of insects, crustaceans, fish, vertebrate animals, and various microbes. Plant-pathogenic oomycetes comprise approximately 200 formal and provisional species of the genus *Phytophthora*, which are arguably the most devastating pathogens of dicotyledonous plants, as well as downy mildew and *Pythium* species (Kamoun, 2003; Yang et al., 2017). Gene transcriptional regulation has been well characterized as an important biological process necessary for successful infection and normal sexual and asexual development in several oomycete plant pathogens (Wang et al., 2011; Ye et al., 2011; Ah-Fong et al., 2019). Genomic and functional analyses have identified several novel promoters (Mcleod et al., 2004; Xiang et al., 2009; Roy et al., 2013), TFs (Xiang and Judelson, 2010; Ye et al., 2013; Pham et al., 2018), and regulatory non-coding RNAs (Jia et al., 2017; Wang Y. et al., 2018), indicating that a number of transcriptional regulatory components and mechanisms are relatively specific to oomycetes in comparison to other eukaryotic organisms; however, detailed analyses are still largely lacking.

In this study, based on our previous comparative genomics study of the DNA-binding domain-containing proteins of various kingdoms, we found that the “plant-specific” RWP-RK family TFs are widely present among the species of Stramenopila, particularly among the oomycetes. Therefore, we systematically identified the RWP-RK proteins in 10 oomycete species for detailed comparisons of the phylogeny, sequences, and secondary structures of the RWP-RK domains of these proteins in oomycetes and plants. We also compared the transcription levels and patterns of the genes in two model oomycete plant pathogens, *Phytophthora sojae* and *Pythium ultimum*, to make preliminary predictions of the biological roles of the RWP-RK proteins in oomycetes.

## Results

### Distribution of the RWP-RK Domain Across Kingdoms

As a preliminary investigation of the RWP-RK proteins in different species, we queried the RWP-RK family (PF02042) profile in the PFAM database. As shown in Supplementary Figure S1, we identified a total of 1823 RWP-RK proteins in 117 eukaryotic species. As expected, the candidates forming the largest group were from the Viridiplantae (1274 proteins in 86 species) with 15 proteins per species on average, and a few candidates were found in the Amoebozoa (14 proteins in 8 species; slime molds). Unexpectedly, the majority of the remaining candidates were from Stramenopila (194 proteins in 18 species), including not only microalgae and brown algae but also oomycetes. In particular, in *Phytophthora*, *Pythium*, and downy mildew genera of oomycetes, each species had 15 candidate RWP-RK proteins on average, as many as in Viridiplantae. Therefore, in addition to their presence in vascular plants, green algae, and slime molds, the range of identified RWP-RK proteins is now expanded to include the Stramenopila, with average family sizes in each oomycete species similar to those in plants.

### Identification of RWP-RK Proteins in Oomycetes

Based on a genome-wide search using HMMER and BLAST programs, and confirmation of candidate proteins using the functional domain prediction servers SMART and NCBI Batch CD-search, we identified 12 to 18 RWP-RK proteins in each of nine oomycete plant pathogens, including *P. sojae* (18), *Phytophthora ramorum* (18), *Phytophthora capsici* (15), *Phytophthora infestans* (15), *Phytophthora parasitica* (16), *Phytophthora litchii* (16), *Hyaloperonospora arabidopsidis* (12), *Plasmopara halstedii* (17), and *Py. ultimum* (15), and identified five RWP-RK proteins in *Saprolegnia parasitica*, which is parasitic to animals (Supplementary Table S1).

After a correction of gene models based on RNA-seq datasets (Supplementary Figure S2) or alignments among orthologs, we found that the lengths of RWP-RK protein sequences ranged from 147 aa to 627 aa, with 313 aa on average, shorter than 672 aa in *Arabidopsis thaliana* and 591 aa in *Glycine max*; in addition, nearly all of the oomycete RWP-RK genes had a single exon, while the majority of the RWP-RK genes in non-oomycetes contained multiple introns (Supplementary Figure S3 and Supplementary Tables S1, S2). In *P. sojae*, only *Ps_139301* might contain an intron; however, it is a likely pseudogene because it had few RNA-seq transcripts and orthologs were absent from many species. In *Py. ultimum*, only *PYU1_T009453* contained an intron; however, it might have alternative splicing with an additional transcript isoform which has no intron (Supplementary Figure S4).

### Phylogenetic Relationships of the RWP-RK Proteins Across Kingdoms

**FIGURE 1:**
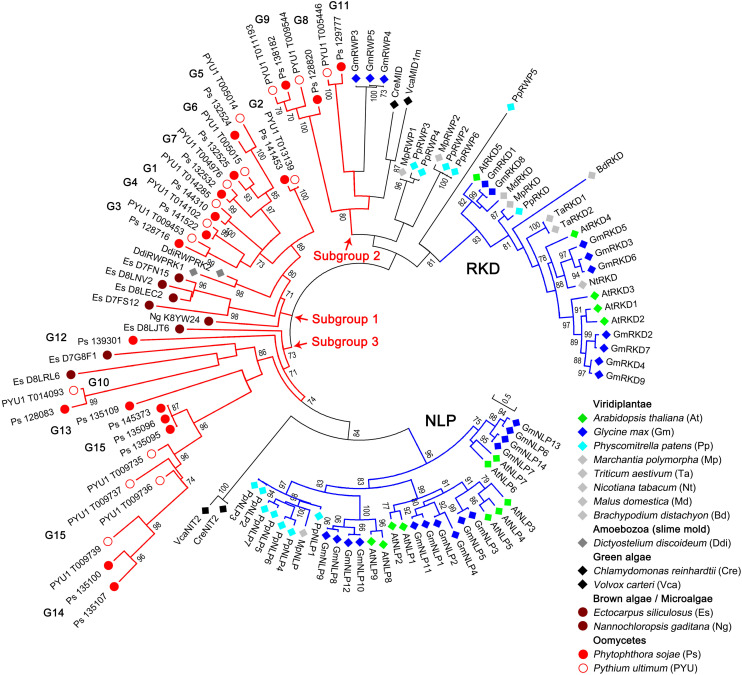
Phylogenetic relationships of the RWP-RK proteins across kingdoms. A phylogenetic neighbor-joining tree was constructed using the RWP-RK domain sequences of the indicated species. Bootstrap values higher than 70 are displayed. The characters in brackets after the species name represent the prefixes of the protein IDs labeled in the tree. G1 to G15 indicate the classified orthologous groups of the oomycete RWP-RK proteins, blue branches indicate the two major sub-families (NLP and RKD) of the plant RWP-RK proteins, and red branches indicate the three major subgroups of the Stramenopila (and Amoebozoa) RWP-RK proteins.

To examine the phylogenetic relationships of the RWP-RK proteins in oomycetes and other species, we constructed a tree using 83 RWP-RK domain sequences, including 33 sequences identified in two representative oomycete species (*P. sojae* and *Py. ultimum*) and 50 sequences from Viridiplantae, Amoebozoa (slime mold), green algae, brown algae, and microalgae (Supplementary Tables S1, S2). As shown in Figure 1, the RWP-RK proteins belonging to the reported RKD and NLP sub-families of Viridiplantae were clearly distinct in the tree, and VcaNIT2 and CreNIT2 of green algae were closely related to the NLP sub-family, while the VcaMID1m and CreMID sequences of green algae, as well as the other proteins of plants (with “RWP” in the IDs; but not Amoebozoa), were closely related to the RKD sub-family.

In addition to these results, which were consistent with previous reports (Koi et al., 2016), we found that the RWP-RK proteins of oomycetes, as well as brown algae, microalgae, and Amoebozoa, were separated from those of Viridiplantae and green algae (Figure 1). According to the topological structure of the tree, the clades for Stramenopila (and Amoebozoa) could be divided into three major subgroups. Subgroups 2 and 3 were more closely related to the RKD and NLP sub-families, respectively, while subgroup 1 was relatively distinct in the tree. Among the three subgroups, the clades of subgroup 1 had relatively short branch lengths and most proteins of brown algae, microalgae, and Amoebozoa were in this subgroup, indicating that evolutionary conservation of RWP-RKs in Stramenopila (and Amoebozoa) was higher in subgroup 1 than in subgroups 2 and 3 (Figure 1).

### Phylogenetic Relationships of the RWP-RK Proteins in Oomycetes

**FIGURE 2:**
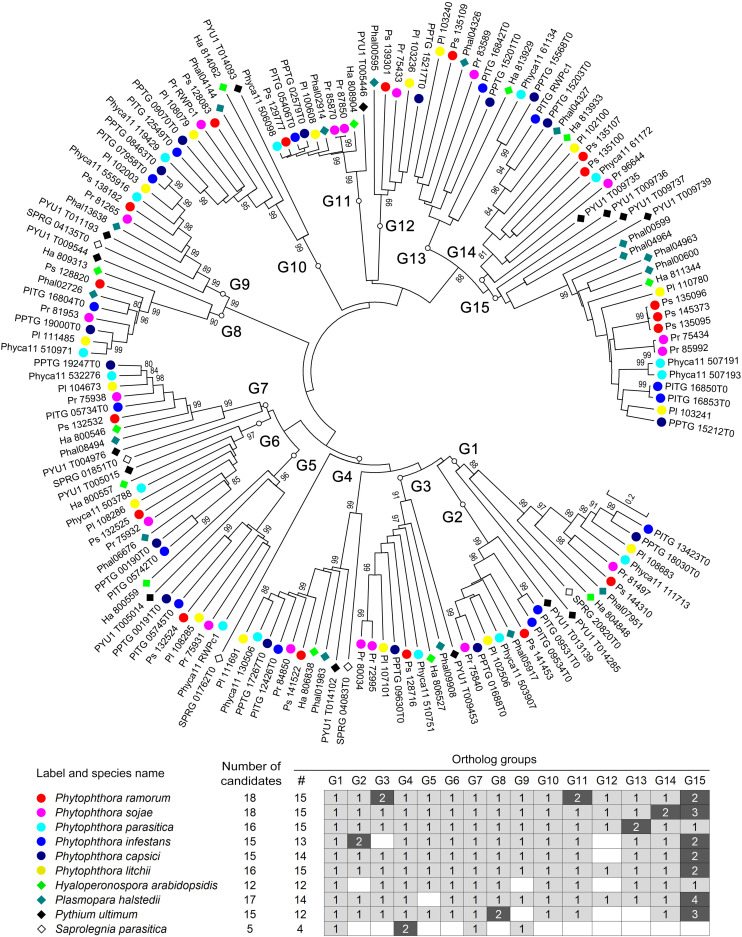
Phylogenetic relationships of the RWP-RK proteins in oomycetes. A phylogenetic neighbor-joining tree was constructed using the RWP-RK domain sequences of the indicated species. Bootstrap values higher than 80 are displayed. G1 to G15 indicate the classified orthologous groups of the oomycete RWP-RK proteins.

For a more detailed understanding of the evolution of RWP-RK proteins in oomycetes, we also constructed a phylogenetic tree using all 147 RWP-RK domain sequences identified from the 10 oomycete species. As shown in Figure 2, there were 15 obvious orthologous groups (termed G1 to G15) among the RWP-RK proteins. The majority of the members in each group were also syntenic with conserved gene order, further confirming their orthologous relationships (Supplementary Figure S5). The majority of the species of the *Phytophthora*, *Pythium*, and downy mildew genera had members belonging to 12 to 15 orthologous groups, while those of *S*. *parasitica* belonged to only four such groups, indicating that the RWP-RK protein family was highly conserved in all of the analyzed oomycete plant pathogens.

Among the 15 orthologous groups of oomycete RWP-RK proteins, six species contained two candidate proteins in one or two specific group(s) of the G1 through G14 groups, e.g., G3 and G11 in *P*. *ramorum*, G14 in *P*. *sojae*, and G2 in *P*. *infestans*; while seven species contained two to four members in G15 (Figure 2 and Supplementary Figure S5). These results may imply later duplication-like events in specific or several oomycete species. In addition, members of the G5–G7 and G13–G15 groups were clustered in the genomes (Supplementary Figure S5) and were likely duplicated in ancient ancestors.

### Prediction of Protein Domains and Additional Motifs

**FIGURE 3:**
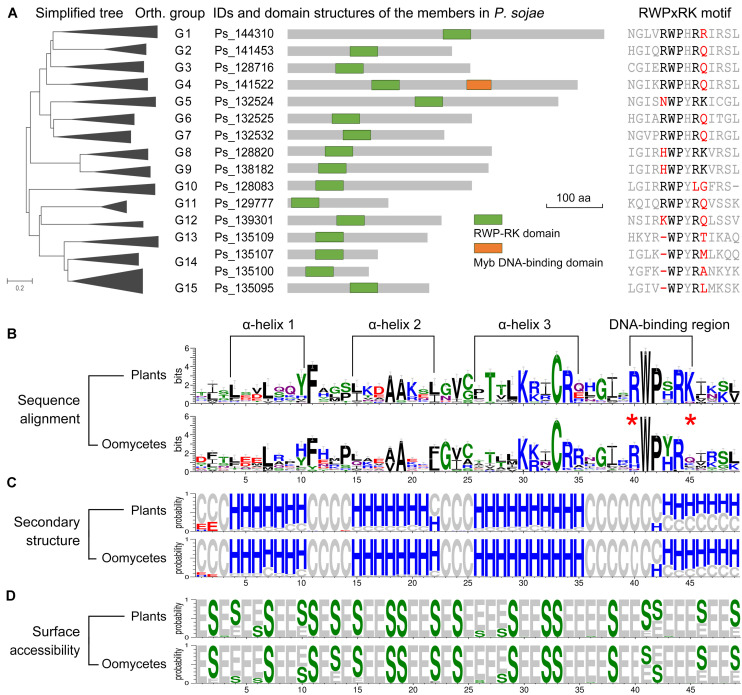
Predicted functional domains of RWP-RK proteins and secondary structures of RWP-RK domains. **(A)** Predicted functional domains of *Phytophthora sojae* RWP-RK proteins and sequence alignment of the corresponding RWPxRK motif. **(B–D)** Weblogo presentation of conservation of the RWP-RK domains in plants and oomycetes. With respect to sequence alignment, the height of symbols within the stack indicates the relative level of conservation of each amino acid at that position. With respect to secondary structure and surface accessibility, “H”, “E”, and “C” represent alpha helix, beta strand, and random coil, respectively; “S” and “E” represent buried and exposed residues, respectively; and the height of symbols within the stack indicates the relative frequency of each result at that position.

The arrangement of the predicted functional domain(s) was conserved in each orthologous group among the oomycete RWP-RK proteins (Supplementary Table S1). The RWP-RK proteins of all groups G1 to G15 contained a single RWP-RK domain, except for the members of G4 (e.g., Ps_141522 of *P. sojae*), which contained an additional Myb DNA-binding domain (Figure 3A). The PB1 or ParM-like domains shared in the RWP-RK proteins of NLP sub-family were not found in any oomycete RWP-RK proteins (Supplementary Figure S6A). Based on MEME webserver^1^ for a *de novo* discovery of conserved motifs in the RWP-RK proteins of *A. thaliana* and *P. sojae*, we identified four motifs additional to those at the RWP-RK domain region only for the NLP sub-family RWP-RK proteins in *A. thaliana* (Supplementary Figure S6B). However, in some ortholog group(s) of oomycetes, the RWP-RK proteins shared specific motif(s) (Supplementary Figure S7).

### Comparison of RWP-RK Domain Secondary Structures

In the sequence alignments of RWP-RK domains, we observed conserved amino acids similar to those of the RWPxRK motif of plants, although “R” at the first position (R^1st^) and “K” at the sixth position (K^6th^) were less conserved (Figure 3A and Supplementary Figure S3). In a comparison between plants and oomycetes, similar conserved amino acids were found not only for the RWPxRK motif itself, but also for the full region of the RWP-RK domain (Figure 3B). The predicted secondary structures contained four conserved alpha helices in the RWP-RK domains of both plants and oomycetes, and the RWP-RK motif was located at the junction of the fourth alpha helix and its upstream coil (Figure 3C). Based on predicted protein surface accessibility, we found that the majority of the conserved amino acids in the RWP-RK domain were buried, which was likely indicative of their role as protein scaffolds (Figure 3D). As a putative DNA-binding region, the most conserved W^2nd^ and P^3rd^ positions of the RWPxRK motif tended to be buried, while the other sites were exposed (Figure 3D). These exposed sites might be located on the protein surface and be associated with DNA-binding affinity.

### RWP-RK Transcription Levels in Oomycete Plant Pathogens

**FIGURE 4:**
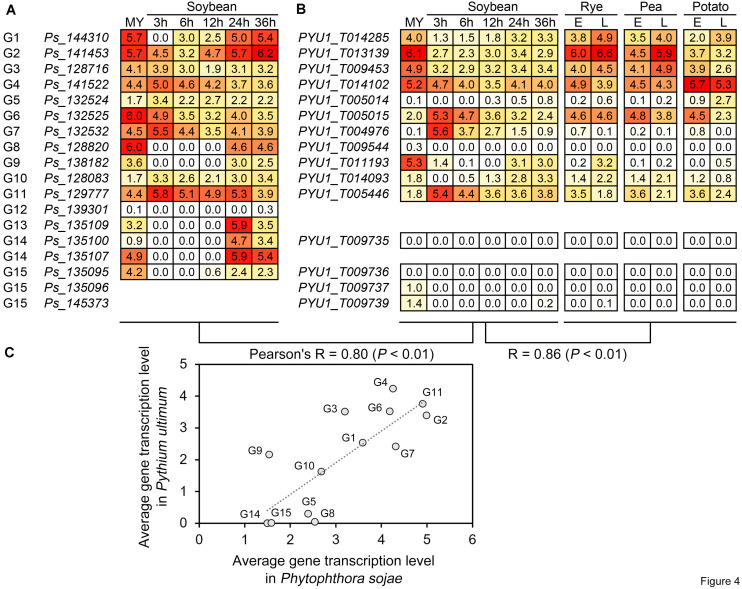
Transcription of the RWP-RK genes in *Phytophthora sojae* and *Pythium ultimum*. **(A)** Transcription of the indicated *Phytophthora sojae* RWP-RK genes in mycelia and at 3, 6, 24, 24, and 36 h post-infection in soybean roots. **(B)** Transcription of the indicated *Pythium ultimum* RWP-RK genes in mycelia and at 3, 6, 24, 24, and 36 h post-infection in soybean hypocotyls, and early and late stages during infection of rye, pea, and potato. The numbers in the blocks represent log_2_ values converted from FPKM, with colored backgrounds from red to white representing the values from high to low. **(C)** Correlation of average transcription levels between orthologous genes or treatments. Average transcription levels were calculated according to the indicated RNA-seq stages or treatments.

Based on the RNA-seq data of *P. sojae* and *Py. ultimum* during host infection, we further compared the transcriptional levels and patterns of the RWP-RK genes to analyze their activity and predict their potential biological roles in oomycete plant pathogens. Under similar treatments, i.e., mycelia and soybean roots or hypocotyls after 3, 6, 12, 24, and 36 h of infection with zoospores, the average transcription levels of the orthologous genes in the two species showed an overall Pearson’s correlation coefficient (*R*) of 0.80, indicating a positive correlation (Figures 4A–C). In general, the overall transcription levels of the RWP-RK genes in subgroup 1 (G1–G7) were relatively higher than those in subgroups 2 and 3 (G8–G15); in both pathogens, the transcription levels of genes in G1–G4, G6, and G11 were relatively higher (Figures 4A–C). In addition to soybean, the *Py. ultimum* RWP-RK genes also showed similar transcription levels during the infection in other hosts (rye, pea, and potato; *R* = 0.86; Figure 4B). The results indicated that the transcription levels of the orthologous RWP-RK genes in oomycete plant pathogens were likely conserved and that some genes were more active transcriptionally while some were nearly silenced.

Several pairs of orthologous genes in the two species exhibited conserved patterns of transcription. For example, in G1 and G9, the RWP-RK genes exhibited transcription levels in both *P. sojae* and *Py. ultimum* that were relatively weaker during the early stage of infection than during the late stage of infection and mycelial stage (Figures 4A,B: soybean, 3—12 h < 24—36 h; rye/pea/potato, E < L), while in G6, G7, and G11, the RWP-RK genes exhibited relatively higher transcription levels during the early stage of infection than during the other stages of infection even mycelial stage (Figures 4A,B: soybean, 3 > 12–36 h; rye/pea/potato, E > L). The results indicated that the transcription of some RWP-RK genes might be repressed or activated to function during specific stages of infection.

## Discussion

In this study, we showed that the well-characterized “plant-specific” RWP-RK family TFs are also present in the kingdom Stramenopila. These results provide further evidence to support the previous inference that the RWP-RK domain is an ancient motif rather than the result of novel evolution in plants, while the absence of this domain in the proteome of some kingdoms, such as the metazoans and fungi, might reflect gene loss later in evolution (Schauser et al., 2005).

Unlike the wide distribution in the Viridiplantae and Stramenopila, only a few RWP-RK proteins have also been identified in the Amoebozoa or slime molds such as *Dictyostelium*, and they were identified as an outgroup of plant RWP-RK domains in a phylogenetic tree (Koi et al., 2016). It has been speculated that they may have been acquired through horizontal gene transfer (HGT) (Schauser et al., 2005). In our results, we found that the two RWP-RK domains in *Dictyostelium discoideum* nested in clades associated with brown algae and oomycetes, which further supports that the donor for HGT of RWP-RK domains to slime molds is likely to have been in the Stramenopila, predating the brown algae and oomycetes divide (Figure 1).

In our phylogenetic analysis, the RWP-RK domains of Stramenopila as well as slime molds formed three subgroups distinct from those of plants. Similar to the majority of the non-NLP sub-family proteins in non-oomycetes, the RWP-RK domain alone was present in almost all of the oomycete RWP-RK proteins including those of subgroup 3, which was more closely related to the plant NLP sub-family. By contrast, additional functional NRD and PB1 domains have been found both up- and downstream, respectively, of the RWP-RK domain in NLPs (Chardin et al., 2014; Mu and Luo, 2019). In oomycetes, the RWP-RK proteins in orthologous group G4 contained an additional Myb DNA-binding domain, which was not found in non-oomycete species. All of these results imply that ancient RWP-RK proteins might have contained a single RWP-RK domain, while the ancient proteins of the NLP sub-family in plants might have evolved the additional domains to increase the functional diversity of this TF family, and likewise for the plant NLPs and oomycete RWP-RK proteins in the G4 group.

The analysis of phylogeny, synteny, sequence alignment, secondary structure, and gene transcription levels revealed that the RWP-RK family is conserved among oomycete species in general. The RWP-RK domain is similar between plants and oomycetes in conserved amino acids and secondary structures, but has amino acid differences that are likely associated with DNA-binding affinity. This result is similar to that of the novel bZIP domains in oomycetes (Ye et al., 2013) and adds evidence to support speculation that many transcriptional regulatory components and mechanisms are relatively specific to oomycetes.

Based on the transcriptomic data of *P. sojae* and *Py. ultimum*, we identified RWP-RK genes with high transcription levels as well as RWP-RK genes that exhibited weak transcription levels or were likely silenced. Some of the identified genes might be associated with infection of soybean and/or other hosts. Therefore, the RWP-RK family may have evolved proteins with conserved functions needed for important biological processes in oomycetes, and the functions of the RWP-RK genes in oomycetes would be an interesting topic for study in the future. For example, whether the oomycete RWP-RK proteins, like those of plants, are also involved in regulation of nitrogen metabolism process, and whether nitrogen metabolism is also associated with pathogenicity of oomycetes. In summary, the results of this study provide new insights for further understanding of the evolution and functions of RWP-RK TFs in specific eukaryotic organisms.

## Materials and Methods

### Sequence Data Collection

RWP-RK gene sequences of the oomycetes *P. sojae* (v1), *P. ramorum* (v1.1), *P. capsici* (v1), *P. infestans* (v1), *P. parasitica* (v1), *P. litchii*, *H. arabidopsidis* (v8.3), *Pl. halstedii* (V1), *Py. ultimum* (V1), and *S. parasitica* (V1) were obtained from the EumicrobeDB database^2^ (Panda et al., 2018). Those of the brown alga *Ectocarpus siliculosus* and the green microalga *Nannochloropsis gaditana* were obtained from the UniProt database,^3^ and the others were from a reported dataset (Koi et al., 2016).

### Identification of RWP-RK Proteins

The distribution of the RWP-RK domain across species was obtained from the profile page of the RWP-RK domain (PF02042) in the PFAM database.^4^ To further identify candidate RWP-RK proteins in specific species, the HMMER program (v3.0)^5^ and Hidden Markov Model (HMM) of the RWP-RK domain (PF02042) obtained from the PFAM database were used for the first stage of a search. The identified candidate proteins became new references for the next stage of the search using the BLASTP program (cut-off: e-value < 1e–8) integrated in SeqHunter software (Ye et al., 2010). All candidate proteins were then checked using the functional domain prediction servers SMART^6^ and Batch CD-search of NCBI.^7^ Finally, gene models of the candidate RWP-RK genes in *P. sojae* and *Py. ultimum* were confirmed or updated according to the alignments with the available RNA-seq transcripts (Ah-Fong et al., 2017; Wang Y. et al., 2018), and the results were used as references for those of the other species. RNA-seq data were visualized using Integrative Genomics Viewer (IGV).^8^ Gene structures were plotted using GSDS2.0.^9^ Novel sequence motifs were analyzed using MEME server (see text footnote 1).

### Phylogeny, Synteny, and Protein Structure Analyses

Based on alignments of the RWP-RK domain sequences (Supplementary Tables S1, S2), phylogenetic trees were constructed using MEGA7 software (Kumar et al., 2016) employing the neighbor-joining method; each tree was tested by bootstrapping of 1000 repetitions. Synteny of the RWP-RK genes among oomycetes was analyzed using the web server Oomycete Gene Order Browser (OGOB).^10^ Protein secondary structure and surface accessibility were predicted using NetSurfP-2.0 software.^11^ Conservation of sequence, secondary structure, and surface accessibility was graphically presented using Weblogo3 web server.^12^

### Gene Transcription Analysis

Transcription levels of RWP-RK genes were obtained from published RNA-seq data (Jing et al., 2016; Ah-Fong et al., 2017) (BioProject IDs: PRJNA318321 and PRJNA407960). Those of *P. sojae* were analyzed in mycelia and at 3, 6, 24, 24, and 36 h post-infection in soybean roots. Those of *Py. ultimum* were analyzed in mycelia and at 3, 6, 24, 24, and 36 h post-infection in soybean hypocotyls and in early and late stages during infection of rye, pea, and potato. The presented data on transcription levels in Figure 4 are the log_2_ values converted from fragments per kilobase per million mapped reads (FPKM). The Pearson’s correlation coefficient (*R*) and *P*-value were calculated based on pairs of average gene transcription levels using SPSS Statistics software (v. 26 for Windows; IBM, Armonk, NY, United States).

## Data Availability Statement

All datasets presented in this study are included in the article/Supplementary Material.

## Author Contributions

WY, YW, and XZ conceived the study. MY, ZZ, MX, and WY did the bioinformatics analysis. HF did the transcription analysis. WY and MY wrote the manuscript. All authors contributed to the article and approved the submitted version.

## Conflict of Interest

The authors declare that the research was conducted in the absence of any commercial or financial relationships that could be construed as a potential conflict of interest.

## Footnotes

1 http://meme-suite.org

2 http://www.eumicrobedb.org/eumicrobedb/index.php

3 https://www.uniprot.org

4 https://pfam.xfam.org

5 http://hmmer.org

6 https://smart.embl-heidelberg.de

7 https://www.ncbi.nlm.nih.gov/Structure/bwrpsb/bwrpsb.cgi

8 https://igv.org

9 http://gsds.gao-lab.org/index.php

10 https://ogob.ie

11 http://www.cbs.dtu.dk/services/NetSurfP

12 http://weblogo.threeplusone.com

## Supplementary Material

FIGURE S1 Distribution of RWP-RK proteins across kingdoms. The result is displayed according to the profile of RWP-RK domain (PF02042) in the PFAM database.

FIGURE S2 Correction of RWP-RK gene model using RNA-seq data. Two *Phytophthora sojae genes* are shown as examples. Blue stripes and red stripes represent the original and corrected gene exons, respectively. Gray and bright blue thin lines represent the transcript reads and splicing junctions within transcript reads, respectively.

FIGURE S3 Comparison of gene structures and RWP-RK motif. The tree is consistent with those in Figure 1 for comparison. Wide and thin lines represent exons and introns, respectively.

FIGURE S4 Alignment of RNA-seq reads for *PYU1_T009453*. Blue stripes and blue lines represent the original gene exon and intron, respectively. Gray and bright blue thin lines represent the transcript reads and splicing junctions within transcript reads, respectively.

FIGURE S5 Synteny of the RWP-RK genes in the indicated species. Results of analysis using the Oomycete Gene Order Browser (https://ogob.ie). The IDs of syntenic genes are highlighted with a specific color, and each color represents a group of syntenic genes.

FIGURE S6 Predicted domains **(A)** and motifs **(B)** of the *Arabidopsis thaliana* and *Phytophthora sojae* RWP-RK proteins. Seven motifs were predicted using MEME, and among them, motifs 1-2-3 correspond to the region of RWP-RK domain and motif 4 corresponds to the partial region of PB1 domain, while the others are novel.

FIGURE S7 Predicted motifs of the oomycete RWP-RK proteins in groups G1-G15. Eight motifs were predicted using MEME, and among them, motifs 4-1-2 correspond to the region of RWP-RK domain, while the others are novel.

TABLE S1 Predicted RWP-RK domain-containing proteins in oomycetes.

TABLE S2 RWP-RK domain-containing proteins in non-oomycete species.
