# Supplementary figures and images for: Conserved Subgroups of the Plant-Specific RWP-RK Transcription Factor Family Are Present in Oomycete Pathogens
Source: Front Microbiol. 2020 Jul 28;11:1724. doi: 10.3389/fmicb.2020.01724 (PMC7399023; doi:10.3389/fmicb.2020.01724)

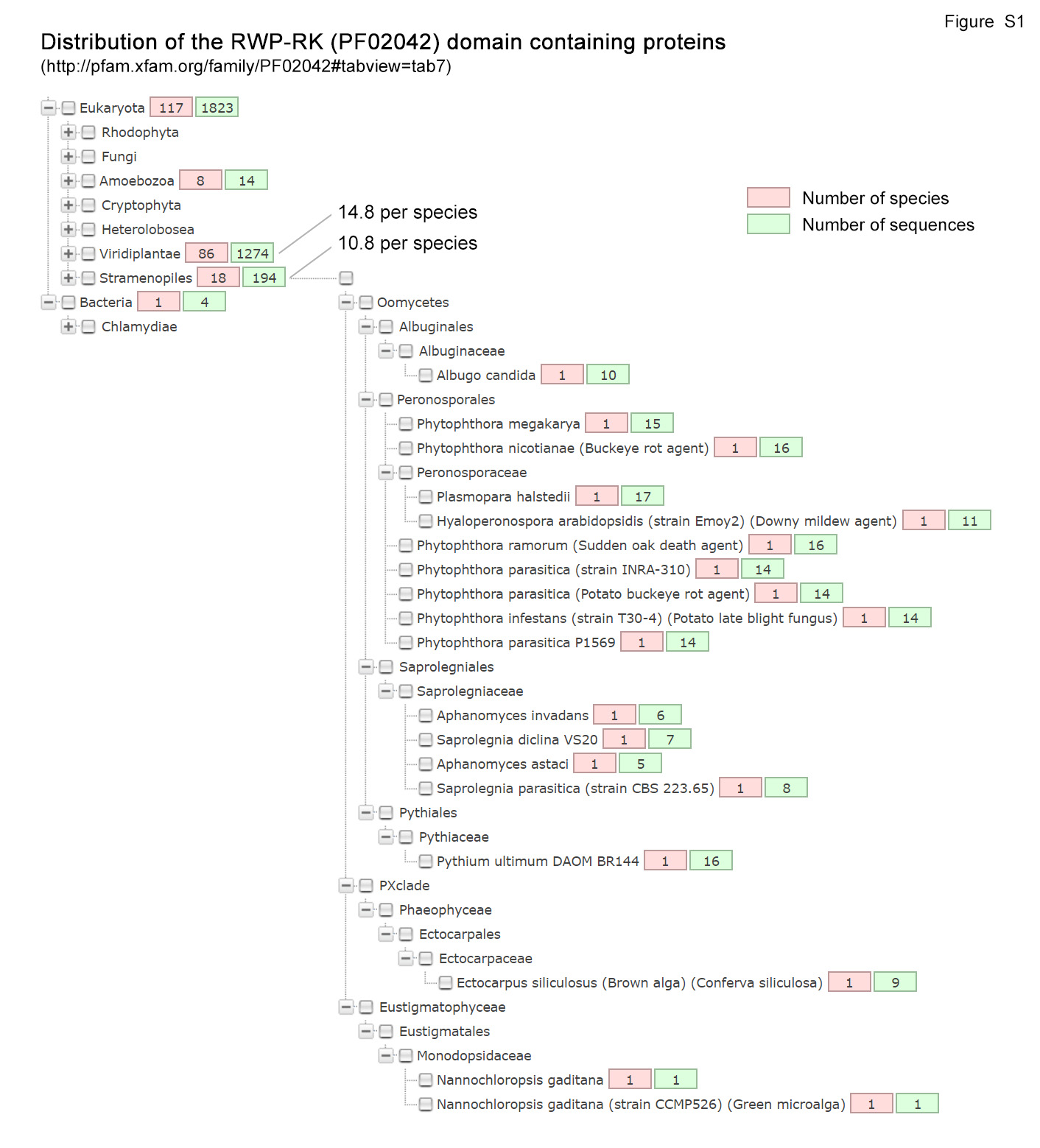

Supplement: FIGURE S1 — Distribution of RWP-RK proteins across kingdoms. The result is displayed according to the profile of RWP-RK domain (PF02042) in the PFAM database. [file Image_1.JPEG]

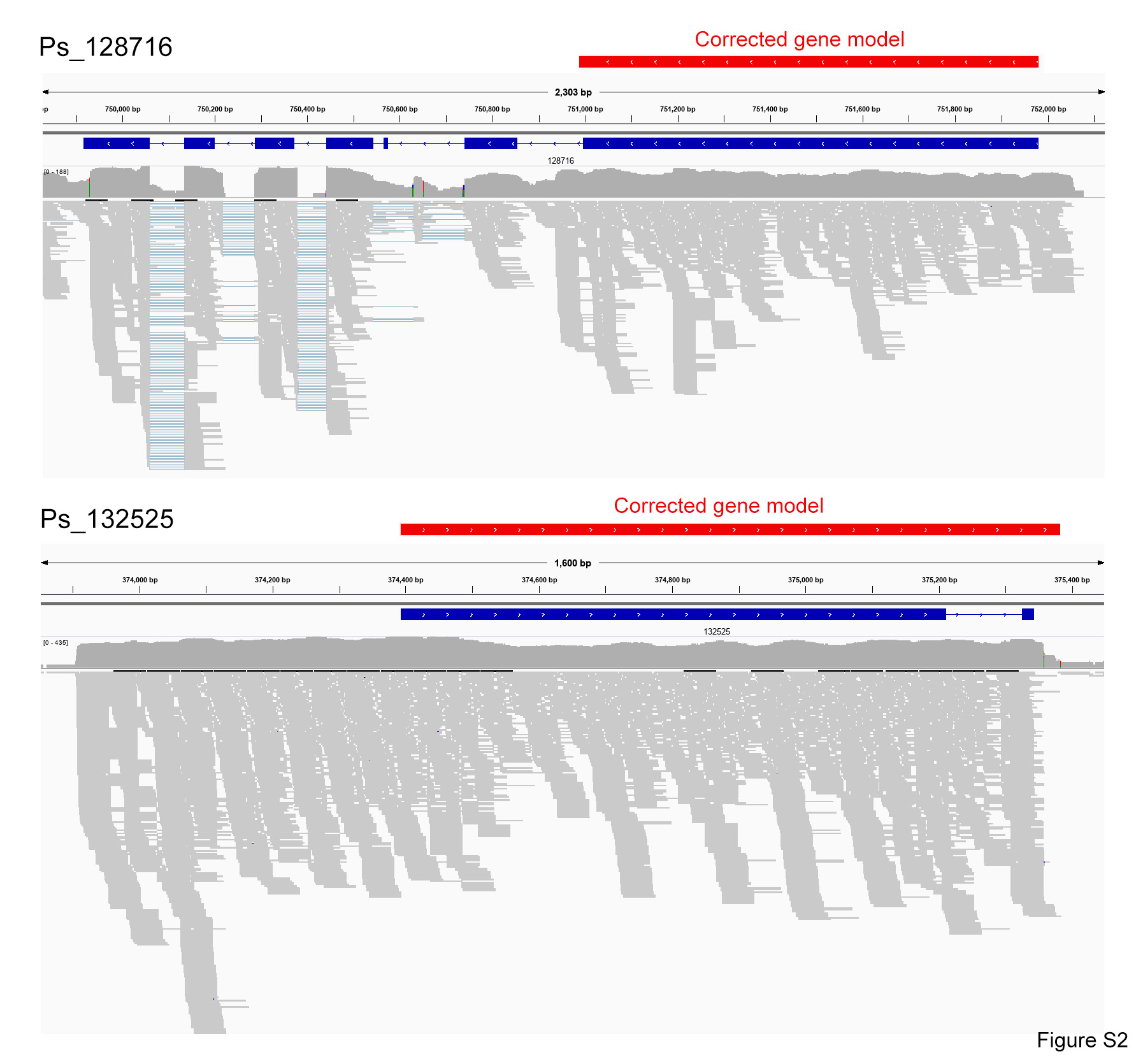

Supplement: FIGURE S2 — Correction of RWP-RK gene model using RNA-seq data. Two Phytophthora sojae genes are shown as examples. Blue stripes and red stripes represent the original and corrected gene exons, respectively. Gray and bright blue thin lines represent the transcript reads and splicing junctions within transcript reads, respectively. [file Image_2.JPEG]

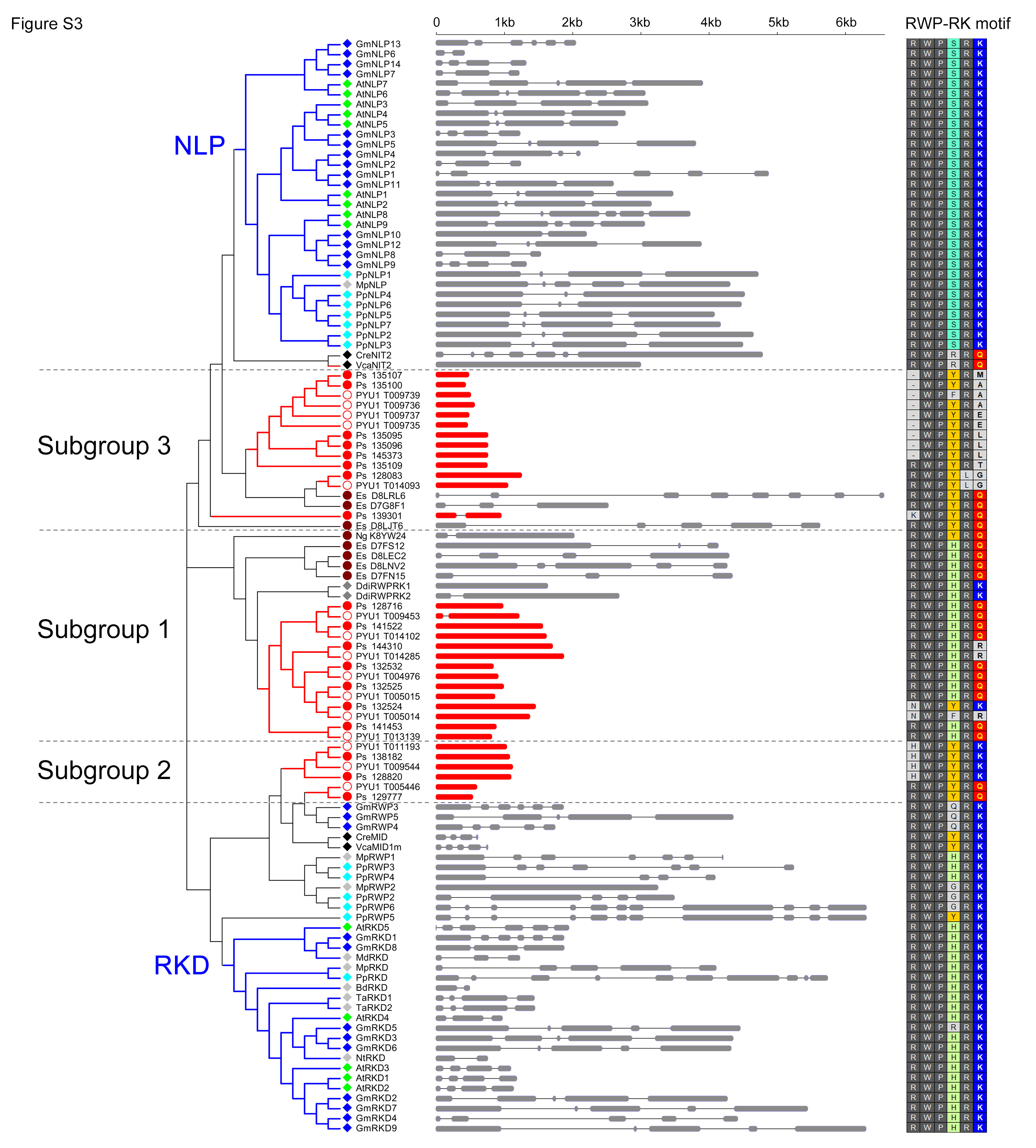

Supplement: FIGURE S3 — Comparison of gene structures and RWP-RK motif. The tree is consistent with those in Figure 1 for comparison. Wide and thin lines represent exons and introns, respectively. [file Image_3.JPEG]

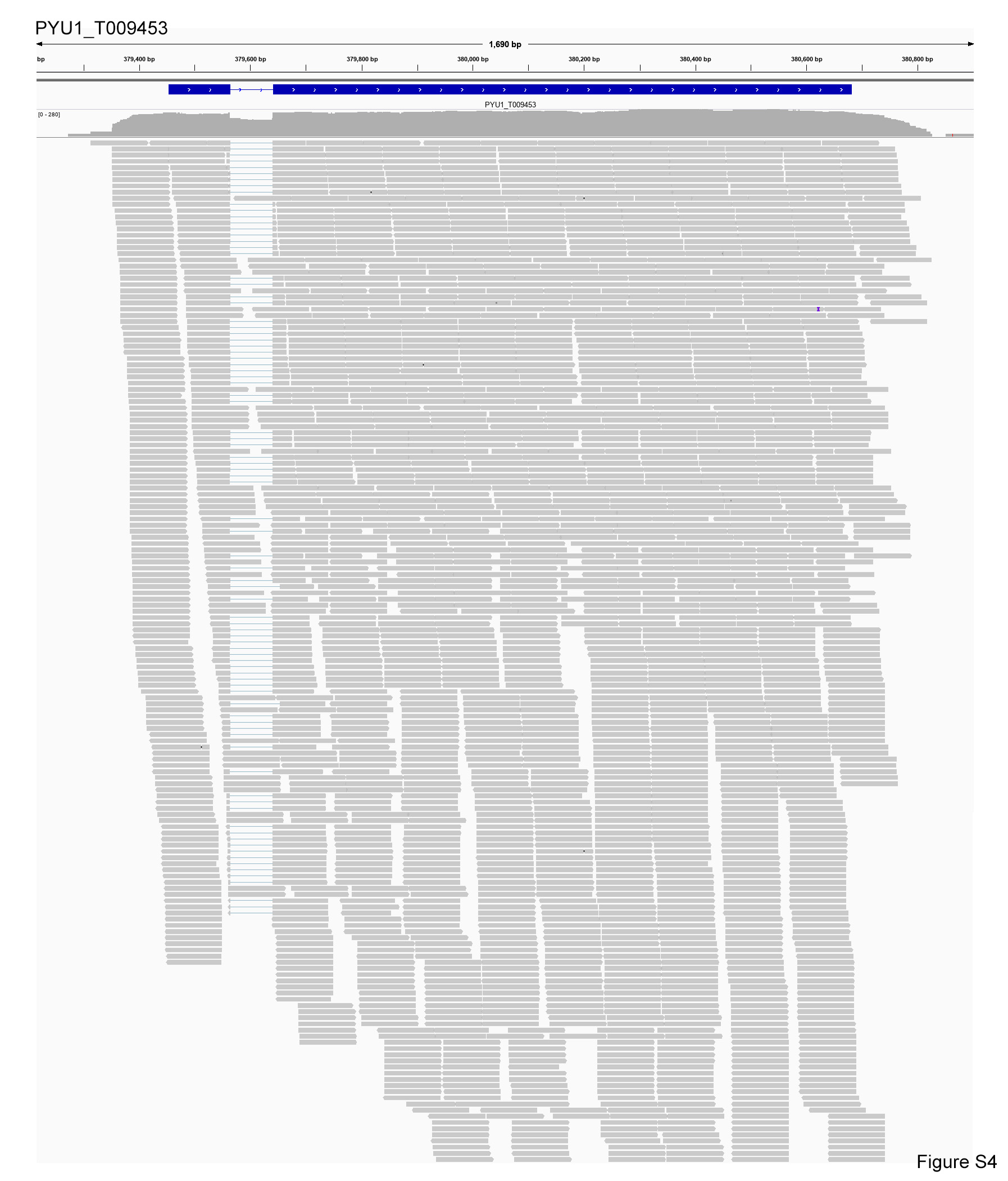

Supplement: FIGURE S4 — Alignment of RNA-seq reads for PYU1_T009453. Blue stripes and blue lines represent the original gene exon and intron, respectively. Gray and bright blue thin lines represent the transcript reads and splicing junctions within transcript reads, respectively. [file Image_4.JPEG]

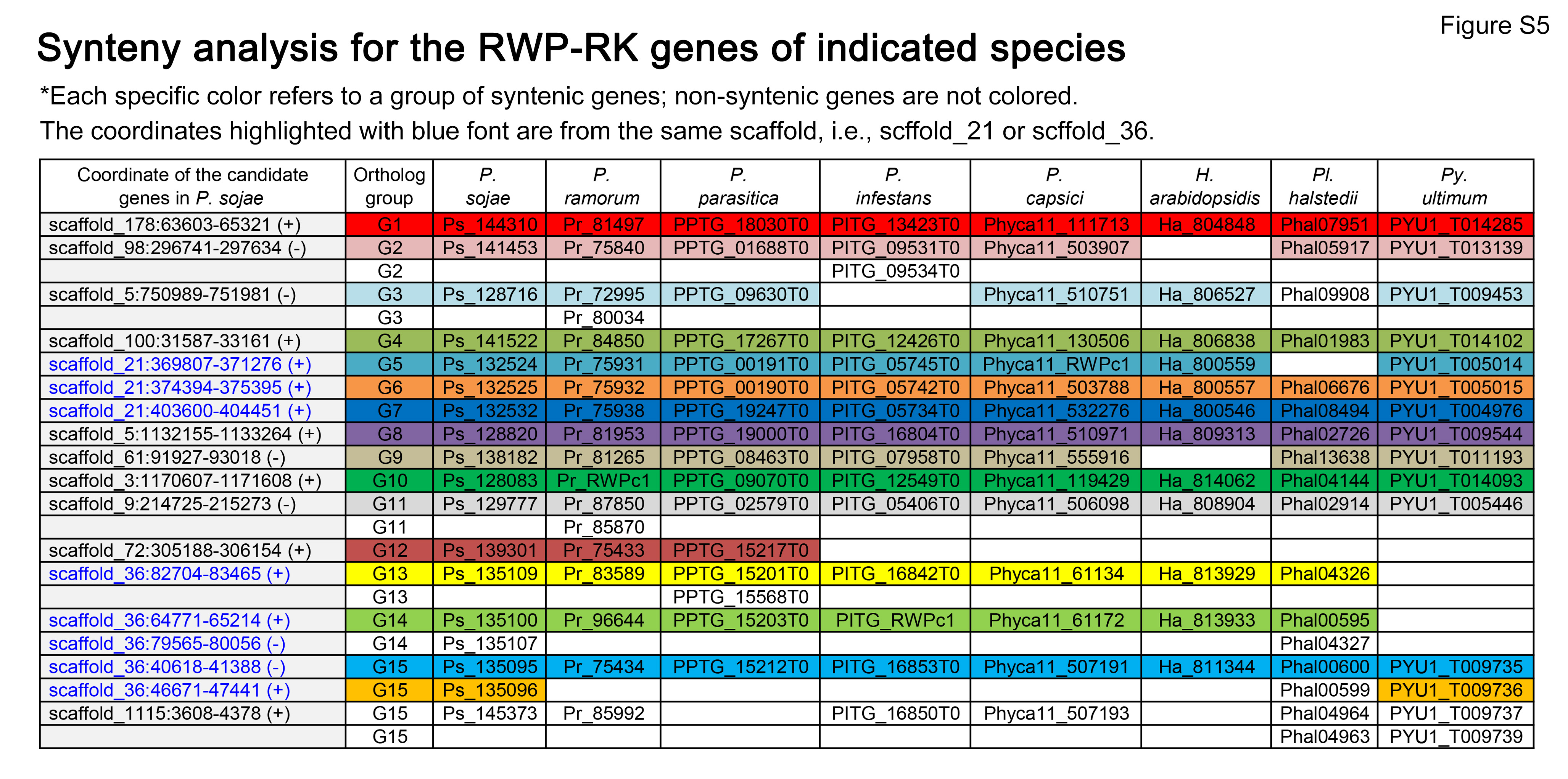

Supplement: FIGURE S5 — Synteny of the RWP-RK genes in the indicated species. Results of analysis using the Oomycete Gene Order Browser (https://ogob.ie). The IDs of syntenic genes are highlighted with a specific color, and each color represents a group of syntenic genes. [file Image_5.JPEG]

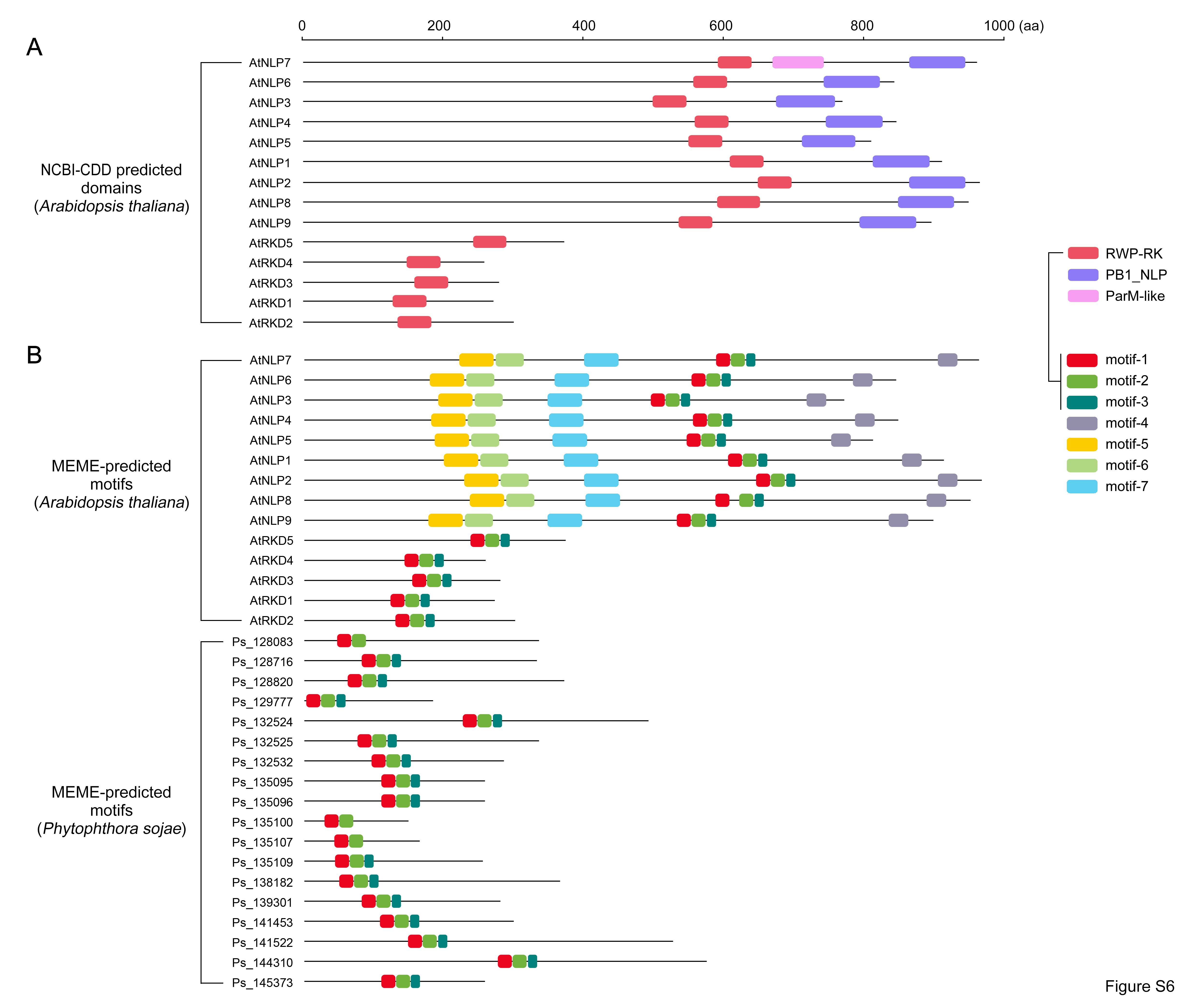

Supplement: FIGURE S6 — Predicted domains (A) and motifs (B) of the Arabidopsis thaliana and Phytophthora sojae RWP-RK proteins. Seven motifs were predicted using MEME, and among them, motifs 1-2-3 correspond to the region of RWP-RK domain and motif 4 corresponds to the partial region of PB1 domain, while the others are novel. [file Image_6.JPEG]

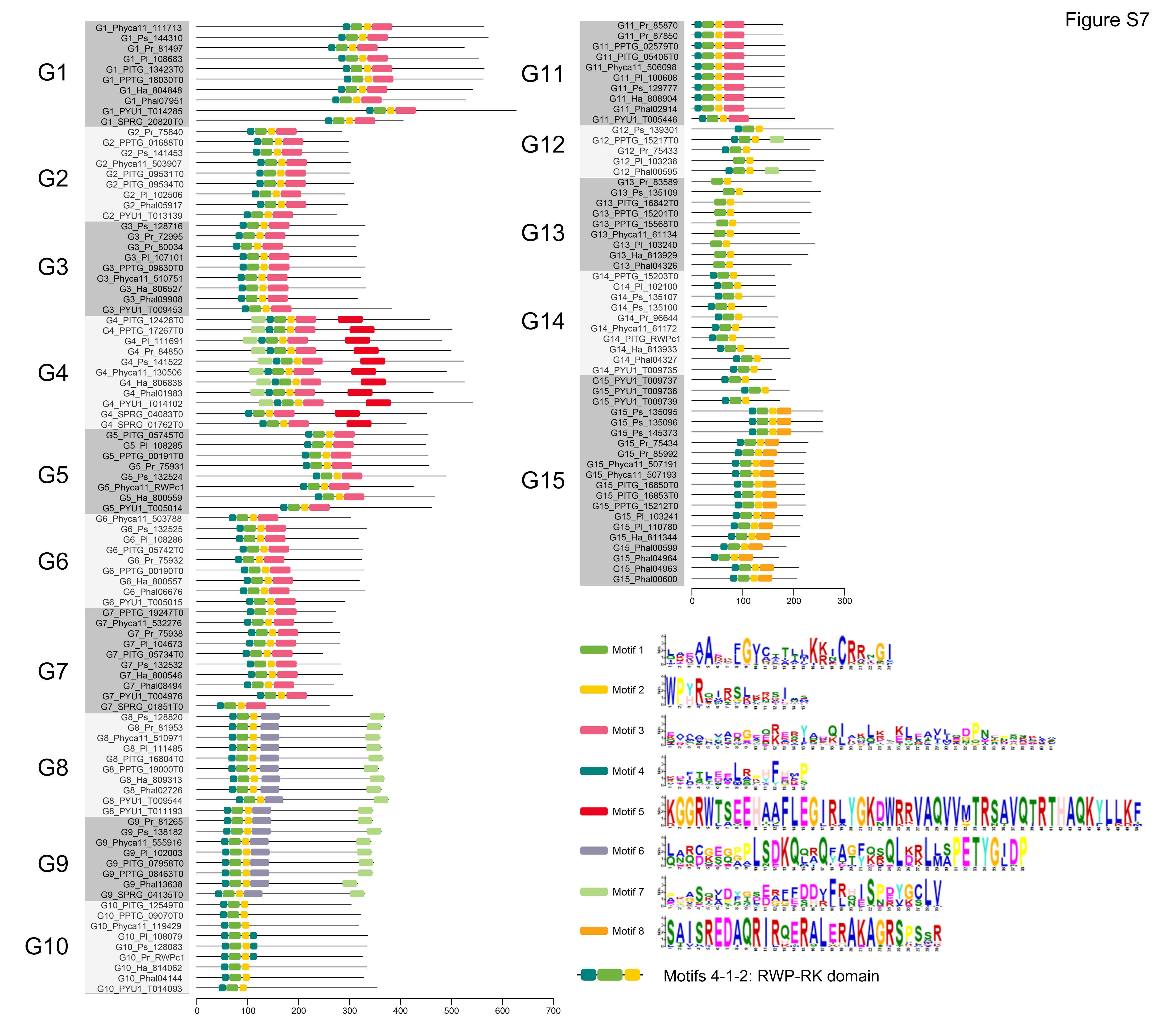

Supplement: FIGURE S7 — Predicted motifs of the oomycete RWP-RK proteins in groups G1-G15. Eight motifs were predicted using MEME, and among them, motifs 4-1-2 correspond to the region of RWP-RK domain, while the others are novel. [file Image_7.JPEG]
